# Early vigour improves phosphate uptake in wheat

**DOI:** 10.1093/jxb/erv403

**Published:** 2015-08-28

**Authors:** Peter R. Ryan, Mingtan Liao, Emmanuel Delhaize, Gregory J. Rebetzke, Chandrakumara Weligama, Wolfgang Spielmeyer, Richard A. James

**Affiliations:** CSIRO Agriculture, GPO Box 1600, Canberra ACT 2601, Australia

**Keywords:** Early vigour, phosphate uptake, phosphorus, QTL, *Rht* genes, wheat.

## Abstract

Quantitative trait loci (QTLs) for shoot biomass were identified in wheat grown on a soil high in total phosphorus (P) but low in plant-available P. The two populations screened included recombinant inbred lines (RILs) from Chuan-Mai 18/Vigour 18 and doubled-haploid lines from Kukri/Janz. Glasshouse-grown plants were harvested at the five-leaf stage. Seven QTLs for shoot biomass were identified in the RILs, with the largest on chromosome 7A accounting for 7.4% of the phenotypic variance. RILs from the upper tail had larger embryos than RILs from the lower tail. Tail lines were then grown in non-limiting P and the results indicated that early vigour and the capacity to access P contributed to the initial distribution. The influence of early vigour on P nutrition was examined further with advanced vigour lines (AVLs). The AVLs accumulated more shoot biomass, maintained lower shoot P concentrations, and showed greater P-acquisition efficiency than Vigour 18. Nine QTLs for shoot biomass were identified in the Kukri/Janz population. Two on chromosomes 4B and 4D accounted for 24.8% of the variance. Candidates underlying these QTLs are the *Rht* genes. We confirmed the influence of these genes using near-isogenic lines with different *Rht* alleles. The dwarf and semi-dwarf alleles affected shoot and root biomass at high and low P but not the efficiency of P acquisition. We conclude that early vigour contributed to the distributions in both populations. Early vigour can increase plant growth at suboptimal P and some sources can also improve the efficiency of P acquisition.

## Highlight

Early vigour in wheat was shown to be a valuable trait for improving biomass accumulation and phosphate uptake from a low-P soil.

## Introduction

Phosphorus (P) fertilizer is essential for sustainable crop production. Most commercial fertilizer is refined from rock phosphate mined from a few high-grade deposits in Africa, China, and the USA (Cordell *et al.*, 2009), and these limited resources need to be used more efficiently in agriculture (Vance *et al.*, 2003; Schroder *et al.*, 2011; Simpson *et al.*, 2011). The adoption of more P-efficient crops and pastures is one strategy to reduce wastage and minimize environmental contamination (Veneklaas *et al.*, 2012).

Phosphorus efficiency can been defined in different ways depending on the spatial and temporal scale being considered. Some definitions refer to yields per unit P applied to a farm or paddock, while others focus on individual plots, biomass accumulation, and even tissue and cell responses. These scales emphasize specific steps of the P cycle to help investigators understand the mechanisms involved (Batten, 1992; Ramaekers *et al.*, 2010; Richardson *et al.*, 2011; Simpson *et al.*, 2011; Bovill *et al.*, 2013). Definitions of P efficiency in plants usually target two fundamental processes: the absorption of P from the soil, which relates to P-acquisition efficiency or P-uptake efficiency, and the conversion of absorbed P into harvestable product (biomass, grain, and fruit), which relates to P-ultilization efficiency. P-efficient crops would ideally utilize all or most of the P applied each season (Helyar, 1998) but this rarely occurs because P can be sequestered into sparingly soluble fractions in the soil, which are unavailable to plants. The presence of these fractions limits P uptake by retarding the replenishment of the soluble-P pool accessed by plants (Richardson *et al.*, 2011). Repeated applications of P fertilizer over years can raise the total soil P level and saturate the reactions that remove P from the soluble pool. Once this occurs, the efficiency of P utilization can improve, but this strategy represents a major long-term investment for farmers (Simpson *et al.*, 2011). Plants that can readily absorb P from the soluble pool and access more of the sparingly soluble P should maintain high yields with reduced inputs.

Plants respond to reduced P availability by changing biomass allocation between roots and shoots, by altering root structure and physiology, and by modifying cellular metabolism (Lopez-Bucio *et al.*, 2002; Vance *et al.*, 2003; Plaxton and Tran, 2011). These responses work to improve P uptake from the soil or increase internal P-use efficiency through better recycling and nutrient distribution (Rose *et al.*, 2013). The significant genotypic variations in these responses can help identify quantitative trait loci (QTLs) for certain adaptive traits, and previous studies have linked some of these to root architecture and function (Batten *et al.*, 1984; Jones *et al.*, 1989; Batten, 1992; Jones *et al.*, 1992; Gahoonia and Nielsen, 1996; Manske *et al.*, 2000; Osborne and Rengel, 2002; Su *et al.*, 2006; López-Arredondo *et al.*, 2014). For example, QTLs for P efficiency have been linked with shallow root angles and with aerenchyma formation in crop species including common bean, soybean, rice, maize, and *Brassica napus* (Zhu *et al.*, 2005; Beebe *et al.*, 2006; Ochoa *et al.*, 2006; Shimizu *et al.*, 2008; Yang *et al.*, 2011; Gamuyao *et al.*, 2012). Similarly, a major QTL for P efficiency in rice is linked to root development. The gene underlying this QTL is *PSTOL1* (*PHOSPHORUS STARVATION TOLERANCE 1*), which controls crown root initiation in seedlings (Wissuwa *et al.*, 2002; Gamuyao *et al.*, 2012).

Most P absorbed by wheat (*Triticum aestivum* L.) occurs pre-anthesis, and early biomass remains a useful surrogate for P uptake and provides a convenient screen for efficiency (Gregory *et al.*, 1979; Grant *et al.*, 2001; Manske *et al.*, 2002; Osborne and Rengel, 2002; Valizadeh *et al.*, 2003). We previously screened 190 genotypes of wheat for shoot biomass in a soil high in total P but low in plant-available P. A selection of contrasting lines was examined in more detail and two genotypes, Kukri and Vigour 18, consistently accumulated greater shoot and root biomass and absorbed more P in low- and high-P treatments than two other genotypes, Janz and Chuan-Mai 18 (Liao *et al.* 2008). Kukri and Vigour 18 also showed greater P-acquisition efficiency (PAE) than Janz and Chuan-Mai 18, which is defined as the ratio of shoot biomass in low P and high P. The present work extends that study by identifying QTLs for early biomass production in low-P soil using a recombinant inbred population from Chuan-Mai 18 and Vigour 18 and a doubled-haploid population from Kukri and Janz. We concluded that early vigour can increase plant growth at suboptimal P and that some sources can also improve the efficiency of P acquisition.

## Materials and methods

### Germplasm

#### Segregating populations.

A family of recombinant inbred lines (RILs) was developed by crossing the Chinese wheat Chuan-Mai 18 with Vigour 18 (male parent). Vigour 18 is a tall line derived from parents Jing Hong 5 and Kharchia and has the wild-type *DELLA* alleles of *Rht-B1a* and *Rht-D1a.* Vigour 18 was generated from an F_2_-derived bulk and so there is some genetic variability among individuals. Chuan-Mai 18 is semi-dwarf line carrying the gibberellin-sensitive dwarfing gene *Rht8* on chromosome 2DS (Botwright *et al.*, 2005). An F5 family was developed by single seed descent for subsequent genotyping. A genetic linkage map was generated with simple sequence repeat markers as described previously (Spielmeyer *et al.*, 2007). The set of doubled-haploid lines (DHLs) was generated using parental cultivars Kukri and Janz as described previously (Kammholz *et al.*, 2001).

#### Advanced vigour lines (AVLs).

Cycle 4 vigour selections were included in additional pot trials. The AVLs were generated from a recurrent selection programme in which 28 vigorous but genetically diverse wheat lines, including Vigour 18, were intercrossed at random. S1:2 progeny were screened, and those with the widest leaves (leaves 1 and 2) were inter-mated and assessed for early growth. This procedure was repeated for six cycles over 15 years. Development of this material has been described in detail elsewhere (Zhang *et al.*, 2015). The AVLs included in this study were named C4_Vig38-19, C4_Vig 37-6c, C4_Vig92-11, and C4_Vig119-4, referred to here as AVL1, AVL2, AVL3, and AVL4, respectively.

#### Near-isogenic DELLA mutants.

Three near-isogenic wheat lines in a Maringa background were homozygous for different *Della* alleles on chromosome 4B. These included *Rht-B1a*, which is the wild-type allele associated with a tall habit (denoted here as NIL_Tall_), the *Rht-B1b* allele associated with a semi-dwarf habit (denoted here as NIL_SD_), and *Rht-B1c* associated with a dwarf habit (denoted here as NIL_DWF_). These alleles were backcrossed seven times into cultivar Maringa by M. D. Gale (Plant Breeding Institute, Cambridge, UK) and then selfed.

### Primary QTL screens

Wheat populations were screened in a highly P-fixing ferrosol from Robertson NSW (34°35′S, 150°36′E). Soil was air dried, passed through a 5mm sieve, and then stored in bins until used for analysis and pot screens. The field capacity of the soil was 37% (Passioura, 2006), the pH was 4.9 (a 1:5 w/v soil:solution using 0.01M CaCl_2_ and 1h shaking), Colwell P was 35mg kg^–1^ of P (Colwell, 1963), and the P buffer index was 1177mg kg^–1^ of P. The high P buffer index was consistent with the high total P content (2400mg kg^–1^) but a low plant-available P (1.3mg kg^–1^ resin extractable) (Burkitt *et al.*, 2002). Total P was determined as described by Olsen and Sommers (1982). P concentrations for the Colwell P and total P assays were determined using the malachite green method.

The experimental design was a complete factorial with four replicates conducted in a naturally lit glasshouse over two sequential growth periods. These were conducted during autumn for Kukri/Janz and spring for Chuan-Mai 18/Vigour 18. Basal nutrients were applied as solutions to 3kg batches of soil in plastic bags at a rate (mg kg^–1^) of 130 N, 50 K, 20 Mg, 20 Ca, 5 Zn, 2 Cu, 2 B, and 1 Mo. The nutrients were allowed to dry and then incorporated into the soil by inverting the bag end over end at least 25 times. P was added to the equivalent of 125kg ha^–1^ as finely ground triple superphosphate (20.7% P; ~0.7g (kg soil)^–1^) immediately before incorporating the other nutrients. This rate of P was shown previously to be growth limiting on this soil (Liao *et al.*, 2008). Plastic bags were placed in the pots (20cm diameter×15cm high), brought to 85% field capacity with deionized water, and left for 10 d to equilibrate before sowing. Seed weights were selected to fall within the 35–40mg range. Two or three seeds were planted in each pot and thinned in the first week to one uniform seedling. By 35 d after sowing (DAS; approx. five-leaf stage), there was no significant correlation between biomass and seed P content (Liao *et al.*, 2008). Tiller number per pot was recorded and the plants were harvested. Shoots were dried at 70 °C for 48h and weighed.

### QTL analysis

Analysis of the Chuan-Mai 18/Vigour 18 population in producing the genetic map has been detailed by Spielmeyer *et al.* (2007), and contained approximately 450 microsatellite markers. Development of the Kukri/Janz genetic map was reported previously (Rebetzke *et al.*, 2007). The QTL analysis was undertaken using spatially adjusted best linear unbiased estimators for each environment, and preliminary interval mapping was undertaken in MultiQTL (Korol *et al.*, 2001). Multiple interval mapping analysis was then undertaken using forward–backward stepwise, multiple linear regression with a probability into and out of the model of 0.05 and a window size set at 10 cM (Korol *et al.*, 2001). Significant thresholds for QTL detection were calculated for each dataset using 1000 permutations and a genome-wide error rate (α) of 0.10 (suggestive) and 0.05 (significant). The resulting genetic model incorporated significant additive genetic effects and their interactions with environment. The locations of genetic effects of individual QTLs were illustrated on maps drawn using MapChart 2.1 (Voorrips, 2002). A 90% confidence interval was obtained for each QTL location using a one-LOD support interval.

### Additional pot trials with Chuan-Mai 18/Vigour 18 RILs

Two additional pot experiments were conducted on selected RILs. The first experiment, conducted in spring, used the parental lines and five RILs from the upper and lower tails of the initial biomass screen grown on the same ferrosol with a high-P treatment. Mean weights (±SE) for grain among the RILs from the upper and lower tails were 46.8±3.5 and 42.4±2.6mg, respectively. Pots were filled with 2.7kg of soil amended with a single high-P treatment of 1250mg kg^–1^ of P using finely ground KH_2_PO_4_ (22.8% P). This rate of P allowed non-limiting growth on this highly P-fixing soil (Liao *et al.*, 2008; Ryan *et al.*, 2014). Pots were weighed regularly to maintain moisture at 85–90% field capacity. At 27 DAS, the width of the first and second leaves was measured and shoots were harvested to determine dry weights.

Another experiment tested the role of vigour using AVLs. This experiment was conducted in spring in a red kandosol with a Colwell P of 16.7mg kg^–1^, a pH of 4.42, and a field capacity of 18.1% (Ryan *et al.*, 2014). Air-dried soil was sieved through a 4mm sieve and amended with 0.7g kg^–1^ of lime to raise the pH to 5.5 and two rates of finely ground KH_2_PO_4_ (22.8% P): a low growth-limiting treatment of 5mg kg^–1^ and a high non-limiting rate of 100mg kg^–1^. The soil was brought up to 85% field capacity by mixing with nutrient solution: 6.5mM KNO_3_, 2mM Ca(NO_3_)_2_, 3mM (NH_4_)_2_SO_4_, 2mM MgSO_4_, 0.045mM FeCl_3_ and micronutrients (B, Mn, Zn, Mo, Cu) just prior to packing of pots. Cylindrical pots of 10.5cm internal diameter and 20cm height were filled with 1.8kg of dry soil to a bulk density of 1.35g cm^–3^. Seeds were selected within a 5mg weight range (45–50mg), imbibed for 2h, treated with a fungicide (Thiram, 4mg l^–1^) and germinated on filter paper in Petri dishes over 2 d. Germinated seeds were planted one per pot at a depth of 1cm. The soil surface was covered with a layer of white plastic beads to reduce water evaporation. The plants were grown in a naturally lit glasshouse. The air temperature in the glasshouse was maintained at approximately 24 °C for the day and 12 °C for the night. The soil temperature was maintained at approximately 16 °C by placing pots in a reticulated water-cooling tank system. Pots were watered with deionized water to 85% of field capacity by weight every 2 d.

Shoots were harvested at 29 DAS (approx. five-leaf stage), dried at 70 °C for 48h, and weighed. Dried shoots were milled for determination of total P. The samples (about 50mg) were ignited in a muffle furnace at 550 °C for 5h. Ashed samples were subsequently dissolved in 5ml of 2M HCl and phosphate concentration was determined by the malachite green method. Shoot P content was calculated as the product of shoot dry weight and P concentration in the shoot. Roots were recovered from soil using a gentle water spray on 1mm sieves. Roots were stored in 50% ethanol at 4 °C prior to analysis. Preserved roots were floated on a plastic tray and scanned using a flatbed scanner (Epson Expression 800) at a resolution of 400 dpi. Greyscale root images were analysed for total root length using WINRhizo Pro (version 2002). The roots were then dried at 70 °C for 48h and weighed. WINRhizo Pro measured root diameters, and fine roots were designated as those with diameters of <0.36mm (mainly the laterals and branch root fraction), while thick roots had diameters of ≥0.36 (mainly seminals and nodal roots).

PAE was defined as the ratio of shoot biomass with low-P treatment to shoot biomass with high-P treatment. PAE estimates the relative capacity of plants to access P and accumulate biomass on soil with low levels of available P compared with soils with high levels of P. Phosphorus-uptake efficiency was defined as total shoot P per unit root length (PUE_Length_) or per unit root weight (PUE_Root DW_). The experiments were arranged in a factorial design with two P treatments and six wheat genotypes in four replicate blocks.

### Pot experiments with Maringa near-isogenic lines (NILs)

An experiment was conducted using near-isogenic Maringa lines with either the wild-type *Rht* allele (*Rht-B1a*), the semi-dwarf allele (*Rht-B1b*), or the dwarf allele (*Rht-B1c*). These lines were grown for 28 d in pots with 3kg of ferrosol amended with a high-P treatment (1250mg kg^–1^ of P) or a low-P treatment of 250mg kg^–1^ of P added as finely ground KH_2_PO_4_ (22.8% P). Seed weights were all 35–40mg. Pots were weighed regularly to maintain moisture at 85–90% field capacity. Tillers were counted at 15, 22, and 28 DAS. At harvest, data were collected for shoot dry weight. Roots were washed out and stored for later scanning with WinRhizo for total root length and root diameter. Root designations into fine and thick roots were as described above.

### Estimating embryo size

Embryo size between RILs of the Chuan-Mai 18/Vigour 18 population was estimated with a Leica MZFLIII dissector microscope. The dorsal side of the grain was magnified 2.5-fold and the transverse distance between the ridges surrounding the scutellum was marked and measured with the Leica and an eye-piece graticule.

### Statistical analyses

Unless stated elsewhere, statistical analyses were performed with Genstat 11th edn and the statistical package of SigmaPlot^TM^ version 12.4. Least significant differences were determined at a 95% significance level. Data for *t*-tests and one-factor and two-factor ANOVAs were first tested for normality and for equality of variances, and transformations were performed where necessary. To test whether two ratios, R_1_ and R_2_, were different from one another, we adapted an approach based on overlapping confidence limits described previously (Zhou *et al.*, 2013; Ryan *et al.*, 2014).

## Results

Two wheat populations were grown on a ferrosol high in total P but low in plant-available P and phenotyped for shoot biomass accumulation. One population comprised 166 RILs generated from Chuan-Mai 18 and Vigour 18 and the second population comprised 162 DHLs generated from Kukri and Janz.

### Chuan-Mai 18/Vigour 18 RILs

**Fig. 1.**
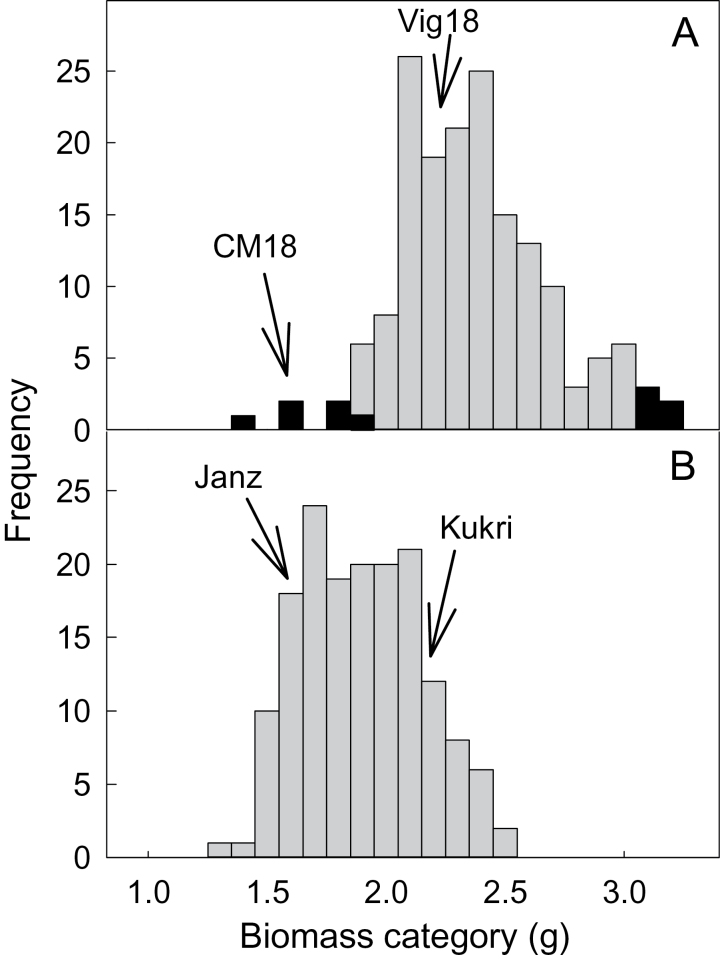
Distribution of shoot biomass for the QTL analyses. The frequency distribution of shoot biomass is shown for the 166 RILs generated from Vigour 18 and Chuan-Mai 18 (A) and 162 DHLs generated from Kukri and Janz (B). The parental lines are indicated in each graph and lines at each tail used in subsequent experiments are coloured black. For each population, the data are the mean of four replicates collected over two experimental runs.

**Fig. 2.**
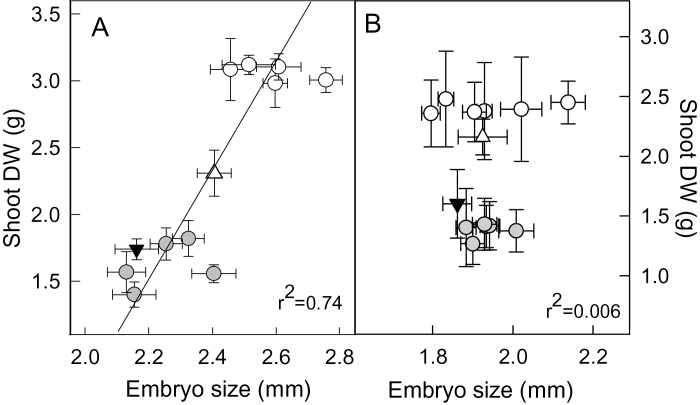
Analysis of tails from the Chuan-Mai 18/ Vigour 18 RILs. (A) Relationship between embryo size and shoot biomass of RILs from the upper tail (open circles) and lower tail (shaded circles) of the Chuan-Mai 18/ Vigour 18 distribution in Fig. 1a. Parental lines Vigour 18 (open triangle) and Chuan-Mai 18 (black triangle) are also included. (B) Relationship between embryo size and shoot biomass of six DHLs from the upper tail (open circles) and lower tail (shaded circles) of the Kukri/Janz distribution in Fig. 1b. Parental lines Kukri (open triangle) and Janz (black triangle) are also included. Results are shown as mean±SE (*n*=12–20 for embryo size; *n*=4 for biomass).

The shoot biomass of the RILs showed transgressive segregation, with half the RILs having a similar or greater biomass than Vigour 18 (Fig. 1A). The ratio of the tails was 0.52±0.03 (see Fig. 2A), which was calculated from the mean biomass of the lower five RILs (1.60±0.06g) divided by the mean biomass of the upper five RILs (3.08±0.02g). There was no significant correlation between shoot biomass and tiller number (*r* ^2^=0.035) over all four replicates. Seven significant QTLs were identified, which collectively accounted for 30% of the phenotypic variation (Table 1, Supplementary Fig. S1, available at *JXB* online). Four QTLs came from Vigour 18, including the largest on chromosome 7A, which accounted for 7.4% of the variance. The remaining QTLs each explained 2.8–4.6% of the variance each. Four additional QTLs were suggestive (significant at *P*<0.1) (Supplementary Table S1, available at *JXB* online).

**Table 1.** QTLs for biomass at low P from the Chuan-Mai 18/Vigour 18 population QTLs were identified by screening 166 RILs on a low-P soil. Shown are the chromosomal locations, nearest linked molecular marker and position, estimated genetic (additive) effects, and the percentage of phenotypic variation explained by the QTL and the LOD score. Positive additive effects indicate that the first parent allele (here Chuan-Mai 18) is associated with increased biomass, whereas a negative effect indicates that Vigour 18 contributed the positive allele. The additive effect ‘*a*’ is estimated as one-half of the difference in homozygotes carrying either parental allele.

Chromosome	Nearest marker	QTL position (cM)^*a*^	*a* genetic effect (%)	Percentage phenotypic variance (σ^2^_P_)	LOD
1B	*wmc44*	108	–0.10	2.8	3.1
3A	*barc045*	35	–0.12	3.3	3.2
3B	*nw3071*	47	0.15	4.3	4.6
4A	*nw2245*	5	–0.14	4.6	4.3
4D	*nw2703*	64	0.13	4.2	3.6
5D	*barc286*	14	0.07	3.5	3.8
7A	*wmc525*	78	–0.17	7.4	5.1

^*a*^ Distance from the tip of the short arm of the chromosome.

Vigour 18 was originally selected for its early vigour. It has larger embryos, wider leaves, and a greater leaf surface area than Chuan-Mai 18 (Richards and Lukacs, 2002; Botwright *et al.*, 2005). We surmised that the distribution in Fig. 1A could have resulted either from: (i) variation in early vigour among the RILs; (ii) variation in the capacity of RILs to extract P from this soil; or (iii) a combination of both. These possibilities were first examined by plotting shoot biomass of the upper and lower RILs against their mean embryo sizes because embryo size is correlated with early vigour in some populations and is independent of treatment effects. The mean embryo size of RILs from the upper tail (2.59±0.05mm, *n*=5) was significantly larger (*P*<0.01) than the average embryo size of RILs from the lower tail (2.25±0.05mm, *n*=5). Embryo size was significantly correlated (*r* ^2^=74; *P*<0.01) with shoot biomass among the tails (Fig. 2A).

These results indicated that early shoot vigour contributed to the variation in Fig. 1A, but it remained unclear whether this was the single major factor or one of many. We reasoned that if vigour was the major determinant of biomass accumulation in Fig. 1A, then a similar variation in biomass should occur when plants are grown at high, non-limiting P. In other words, the biomass ratio of RILs from the tails would be the same in high- and low-P (ratio ~0.5) conditions. If, however, biomass accumulation was driven by other trait(s) that increased the capacity of plants to access P, then all lines should grow to the same size when P is non-limiting. In this case, the biomass of RILs from the lower and upper tails should be similar (ratio ~1.0). If both mechanisms are involved, then the biomass ratio would fall between these values. RILs from the upper and lower tails were grown for 27 d in the same soil amended with a high-P treatment and the null hypothesis was that their biomass ratio would be the same (i.e. early vigour was the major driver of variation in Fig. 1A). The final shoot ratio of the tail lines (lower/upper) was 0.84±0.03, which was significantly greater than the ratio obtained previously at low P (0.52±0.03) but lower than a ratio of 1.0. The null hypothesis was rejected and the results indicated that a combination of early vigour and other factors contributed to the distribution in Fig. 1A. This is consistent with both parental lines donating significant QTLs. Interestingly, RILs from the upper tail also had significantly wider leaves (6.06±0.27) than RILs from the lower tail (5.34±0.16mm) at high P (Supplementary Fig. S2, available at *JXB* online), which provided further evidence that early vigour was an important phenotype in the original screen.

**Fig. 3.**
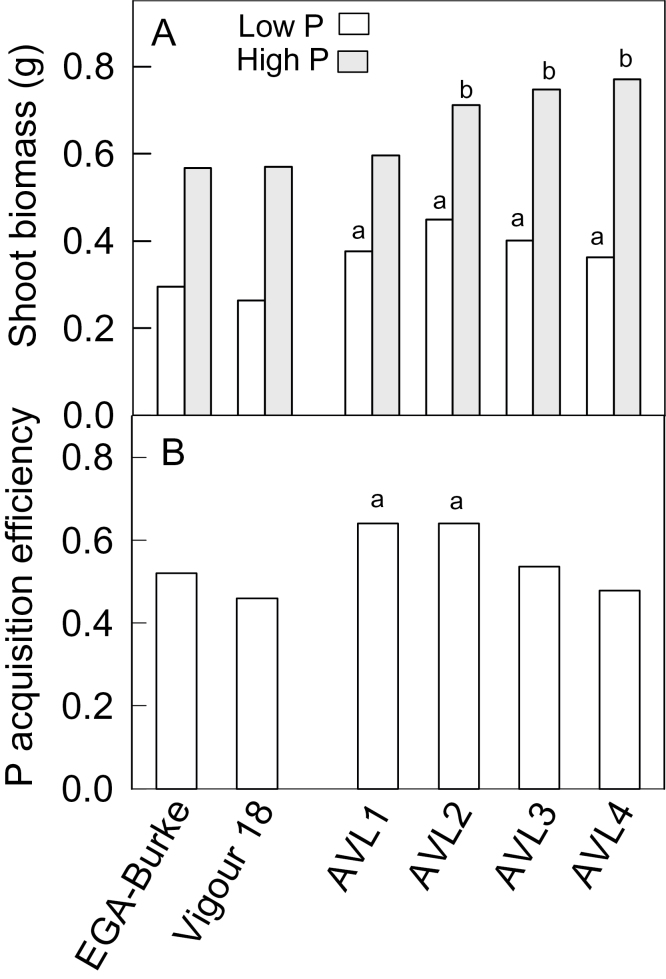
Shoot biomass of the AVLs, Vigour 18 and EGA-Burke. (A) Shoot biomass after 26 d growth in soil amended with P at a low or high rate. Data from the high- and low-P treatments were analysed separately. The least significant difference (*P*<0.05) for the low-P treatment was 0.063 and for high P was 0.088 (*n*=4). AVLs labelled with ‘a’ in the low-P treatment and ‘b’ in the high P treatment had a significantly greater biomass than Vigour 18 and EGA-Burke. (b) PAE, defined as the ratio of shoot biomass at low-P and high-P treatments. AVLs labelled with ‘a’ had a significantly greater PAE than Vigour 18 and EGA-Burke (*P*<0.05).

The above results indicated that a combination of early vigour and other unknown traits contributed to the variation in Fig. 1A. The other traits could be a consequence of vigour (e.g. a larger root system, which acquires more P) or they could be independent of it (e.g. greater internal P-use efficiency, recycling, or changes to root function). We investigated these alternatives using AVLs recently generated from ongoing selections (see Materials and methods). These lines have even wider leaves and show more rapid biomass accumulation than Vigour 18 (Zhang *et al.*, 2015). The embryo sizes of the AVLs were similar to, or larger than, Vigour 18 and EGA-Burke included as a commercial control (Supplementary Fig. S3a, available at *JXB* online). Growth of four AVLs (AVL1, -2, -3, and -4) was compared with Vigour 18 and EGA-Burke on soil amended with high- and low-P treatments. After 26 d, the shoot biomass of all AVLs was ~50% greater than that of Vigour 18 and EGA-Burke in low P (Fig. 3A), and three AVLs had significantly greater biomass than the others in high P. The ratio of shoot biomass at low P and high P is one measure of PAE, and for two AVLs, PAE was significantly larger than Vigour 18 and EGA-Burke (Fig. 3B).

The mechanisms driving the greater PAE of the AVLs were explored by comparing additional shoot and root traits with EGA-Burke. Mean shoot P concentrations were approximately 0.23% dry weight (DW) in low P and 0.63% DW in high P. Three AVLs maintained an ~15% lower P concentration than EGA-Burke in the low P treatment, and one AVL had a lower concentration at high P. Total root biomass (data not shown) and root:shoot ratios (Table 2) were similar in all genotypes at high and low P, but the AVLs had significantly thicker roots than EGA-Burke in low P. Total root length was also similar among the lines in each P treatment, but the AVLs had a lower ratio of finer to thicker roots than EGA-Burke at low P (Table 2). P-uptake efficiency (PUE) was estimated for the lines and this was defined in two ways: total shoot P per unit of root length (PUE_Length_, µg P m^–1^ of root) or total shoot P per unit root biomass (PUE_Root DW_, mg P g^–1^ of DW of root). The PUE_Length_ for three AVLs was significantly greater than that of EGA-Burke at high P and low P, but no differences were detected for PUE_Root DW_ in either treatment (Table 2).

**Table 2.** Comparison of AVLs with EGA-Burke in high- and low-P treatments Shoot and root measurements in AVLs and EGA-Burke grown for 26 d in soil with a low, limiting P rate or high non-limiting P rate. Shown are shoot P concentration, DW ratio of root:shoot, total root length, ratio of total fine-root length (<0.36mm diameter) to total thicker root (≥0.36mm diameter) length, mean root diameters, and PUE expressed as shoot P per unit root length (µg P m^–1^) and per unit root DW (mg P g^–1^ DW). Values are means (*n*=4) and LSD values (*P*<0.05) are provided. The asterisks indicate significant differences compared with EGA-Burke within each P treatment_._

Genotype	Shoot P (% DW)	DW ratio of root:shoot	Total root length (m)	Root length ratio of fine:thick	Root diameter (mm)	PUE_Length_ (µg P m^–1^)	PUE_Root DW_ (mg P g^–1^ DW)
**Low P**							
AVL1	0.249	0.34	37.7	2.80*	0.70*	24.9*	7.5
AVL2	0.227*	0.34	39.3	2.76*	0.69*	26.1*	6.7
AVL3	0.235*	0.32	38.9	2.89*	0.69*	23.9	7.6
AVL4	0.223*	0.29	30.7	2.90*	0.68*	26.4*	7.7
EGA-Burke	0.270	0.38	38.8	3.95	0.59	20.8	7.1
LSD	0.025	NS	NS	0.54	0.05	3.1	NS
**High P**							
AVL1	0.648	0.21	28.8	2.05	0.76	135.4	31.6
AVL2	0.634	0.21	25.7	1.97	0.78	160.4*	29.9
AVL3	0.610	0.21	31.1	2.23	0.87	145.6*	29.2
AVL4	0.564*	0.21	29.9	2.04	0.77	144.6*	26.8
EGA-Burke	0.627	0.21	29.4	2.33	0.72	121.0	29.5
LSD	0.048	NS	NS	NS	NS	22.7	NS

### Kukri/Janz DHLs

Shoot biomass in the Kukri/Janz population grown on the same low-P soil also showed a 2-fold variation (Fig. 1B). The ratio of the tails of the distribution was 0.58±0.01, which was calculated from the mean biomass of the lower five lines (1.37±0.03g) divided by the average biomass of the upper five lines (2.37±0.02g). The correlation between shoot biomass and tiller number across all four replicates was low (*r* ^2=^0.15). The embryo sizes of Kukri and Janz were not significantly different (~1.9±0.1mm), and there was no correlation between embryo size and biomass among the six DHLs from the upper tail and six DHLs from the lower tail (Fig. 2B). Nine significant QTLs for biomass were identified, which accounted for 54.3% of the phenotypic variance. Four of these came from Kukri and five from Janz (Table 3, Supplementary Fig. S4, available at *JXB* online). Two QTLs on chromosomes 4B and 4D alone accounted for 24.8% of the variance. Candidates underlying these two major QTLs are alleles of the *Rht* genes (*Rht-B1b* in Janz and *Rht-D1b* in Kukri), which confer semi-dwarf phenotypes. Two additional QTLs from Janz (chromosome 2D) and Kukri (chromosome 6A) accounted for 7.8 and 7.1% of the phenotypic variance for biomass, respectively, and three QTLs were suggestive (Supplementary Table S1).

**Table 3.** QTLs for biomass at low P from the Kukri/Janz population QTLs were identified by screening 162 DHLs on a low-P soil. Shown are the chromosomal locations, nearest linked molecular marker and position, estimated genetic (additive) effects, and percentage of phenotypic variation explained by the QTL and the LOD score. Positive additive effects indicate that the first parent allele (here Kukri) is associated with increased biomass, whereas a negative effect indicates Janz contributed the positive allele. The additive effect ‘*a*’ is estimated as one-half of the difference in homozygotes carrying either parental allele. Note that Kukri has the tall *Rht* allele (*Rht-B1a*) on chromosome 4B and the semi-dwarf *Rht* allele (*Rht-D1b*) allele on 4D. Janz has the tall *Rht* allele (*Rht-D1a*) on chromosome 4D and the semi-dwarf *Rht* allele (*Rht-B1b*) allele on 4B.

Chromosome	Nearest marker	QTL position (cM)^a^	*a* genetic effect (%)	Percentage phenotypic variance (σ^2^_P_)	LOD
1A	*nw0255*	192	–0.07	2.4	3.2
1B	*cfa2158*	44	–0.05	2.5	2.9
2B	*nw0479*	111	–0.06	2.2	3.1
2D	*gdm006*	271	–0.06	7.8	8.2
4B	*Rht-B1*	11	0.19	17.9	15.3
4D	*Rht-D1*	3	–0.09	6.7	6.2
5A	*gwm304*	33	0.06	2.7	3.2
6A	*nw2119*	68	0.10	7.1	6.7
7B	*wmc276*	93	0.08	4.1	4.7

^*a*^ Distance from the tip of the short arm of the chromosome

We investigated the contribution of the *Rht* alleles on the distribution in Fig. 1B and their influence on PAE and PUE. Growth experiments were performed NILs of wheat with different *Rht* alleles on chromosome 4B. These lines possessed either *Rht-B1a*, the wild-type allele associated with tall plants (NIL_Tall_), the *Rht-B1b* allele, which confers the semi-dwarf habit (NIL_SD_), or the *Rht-B1c* allele, which confers the dwarf habit (NIL_DWF_). No differences were detected in the embryo sizes of these NILs (Supplementary Fig. S3b). Our reasoning for using these NILs was that they are ideal material to independently test whether *Rht* genes influence plant growth on low-P soil. Even though these NILs had a different background (Maringa), they allowed us to directly compare the effect of the *Rht-B1* alleles on P uptake and efficiency in isolation from the other genetic factors.

**Fig. 4.**
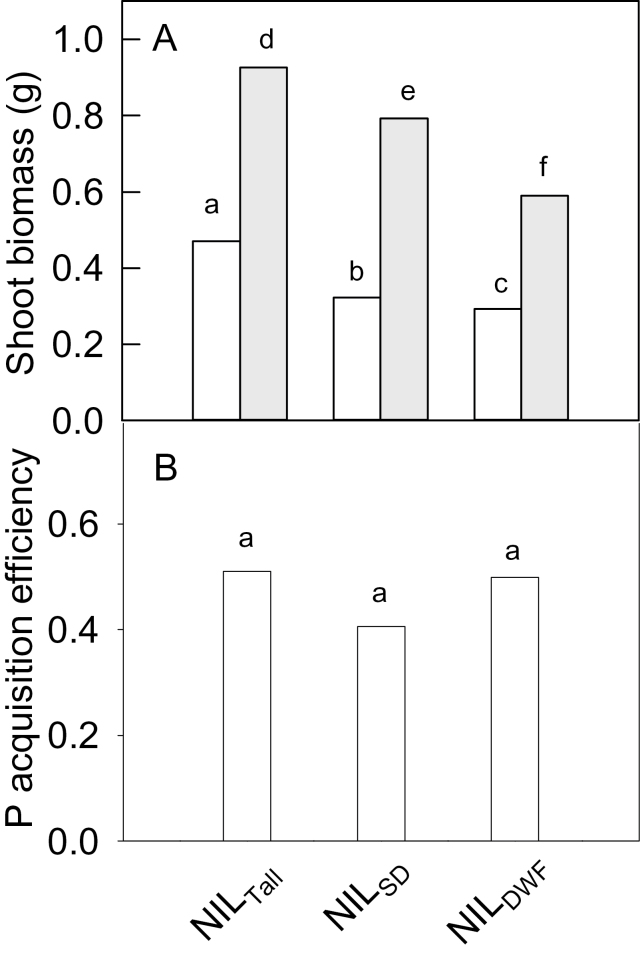
Shoot biomass of NILs differing in dwarfing alleles. (A) Shoot biomass for the three NILs grown in low (white bars) and high (shaded bars) P treatments. NIL_TALL_ is the wild-type *Rht-B1a* allele, NIL_SD_ is the *Rht-B1b* semi-dwarf allele, and NIL_DWF_ is the *Rht-B1c* dwarf allele. Data were transformed with the square root function to satisfy equality of variance and then analysed with a two-factor ANOVA (where genotype and P treatment were factors). Both factors were significant (*P*<0.01), but the interaction was not significant. Data with different letters are significantly different from one another. (b) PAE, defined as the ratio of biomass from the low-P and high-P treatments. Data are means±SE (*n*=6). Columns with different letters are significantly different from one another (*P*<0.05).

Plants were grown in the same soil type with either a low-P treatment (similar to the initial doubled-haploid screen) or a high, non-limiting P treatment. The null hypothesis in these experiments was that the dwarfing genes would affect biomass accumulation at high and low P but would not alter PAE. Fig. 4A shows that the final shoot biomass of NIL_Tall_ plants was significantly greater than that of NIL_SD_ and NIL_DWF_ plants at the high- and low-P treatments. Statistical analysis indicated that there were significant genotype and treatment effects but no significant interactions. PAE was again calculated for each line from the ratio of shoot biomass at low and high P. No statistical differences in PAE were detected between NIL_Tall_, NIL_SD_, and NIL_DWF_ (Fig. 4B), although NIL_SD_ PAE was lower than the others at *P*<0.1. Therefore, the null hypothesis was accepted: the *Rht-B1b* and *Rht-B1c* alleles reduced plant biomass but they did not alter the efficiency of P uptake as measured by PAE. For the low-P treatment, the biomass ratio for NIL_SD_/NIL_Tall_ plants was 0.68±0.07 and the ratio for NIL_DWF_/NIL_Tall_ plants was 0.62±0.05, which was similar to the ratio of the lower and upper tails of the initial doubled-haploid screen (0.58±0.01). This was consistent with the *Rht* genes having a major influence on the distribution in Fig. 1B.

**Fig. 5.**
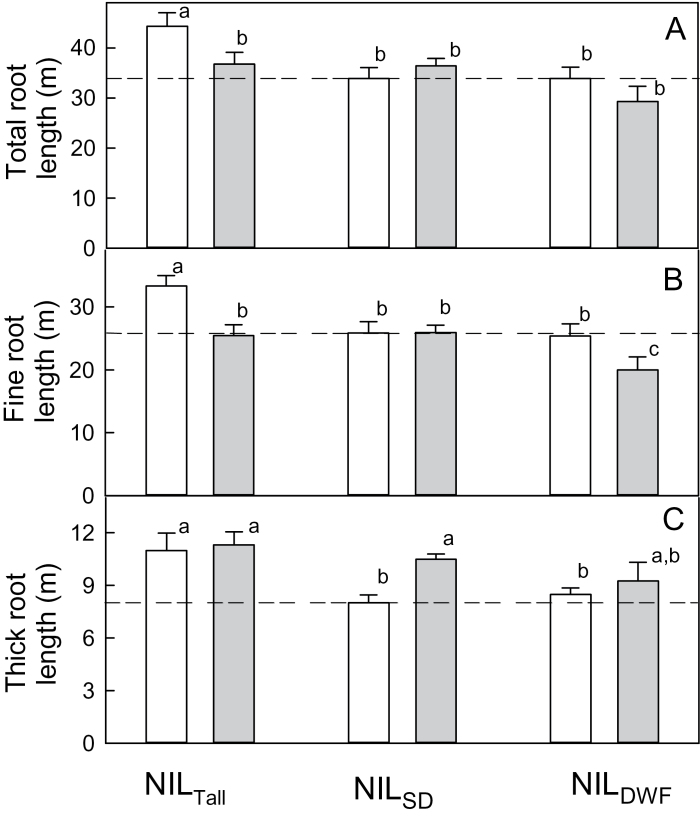
Effect of dwarfing genes on root growth under low and high P. NILs were grown in soil amended with a low-P (white bars) and a high, non-limiting P (shaded bars) treatment. Roots were harvested, washed, and scanned with WinRHIZO. Fine roots were defined as having diameters of <0.36mm and thick roots were ≥0.36mm. Dashed lines are for NIL_SD_ at low P. Data show means±SE (*n*=6). Two-way ANOVA was performed on each set of data. For (A), genotype was significant at *P*<0.01, but P treatment and interactions were non-significant. For (B), genotype was significant at *P*<0.01 and P treatment was significant at *P*<0.05, but the interaction was non-significant. For (C), genotype was significant at *P*<0.01 (NIL_Tall_>NIL_DWF_=NIL_DWF_) and P treatment was significant but the interaction was non-significant. Data with different letters were significantly different from one another.

Tillering could not explain the greater biomass of NIL_Tall_ plants since tillering actually continued for longer in NIL_DWF_ (Supplementary Fig. S5, available at *JXB* online). Total root biomass and the ratio of shoot biomass to root length were not statistically different among the NILs within each P treatment (Supplementary Fig. S6, available at *JXB* online) and neither were root diameters or the root-to-shoot biomass ratio (Table 4). Total root length was the same at high P, but NIL_Tall_ had 30% greater root length at low P due to an increase in fine and thicker root length (Fig. 5). Interestingly, the responses of diameter classes to P differed between the NILs. For NIL_Tall_ and NIL_DWF_, fine-root length increased in low-P compared with high-P treatment (Fig. 5), whereas thick root length was unaffected by P treatment. By contrast, for NIL_SD_ plants, fine-root length was unaffected by P treatment but thick root length was reduced by low P. Despite these changes, the ratio of total fine roots to thicker root lengths was not statistically different between the NILs at either P treatment (Table 4). Shoot P concentrations were ~30% lower in the low-P compared with the high-P treatments, but no significant differences were detected among the lines within each treatment. There were no differences in PUE among the NILs, regardless of whether it was expressed per unit root length (PUE_Length_) or per unit root DW (PUE_Root DW_) (Table 4). The one exception was PUE_Length_ for NIL_DWF_, which was lower than the other NILs at high P.

**Table 4.** Comparison of NILs with contrasting Rht alleles in high- and low-P treatments Shoot and root measurements in NILs grown for 26 d in a ferrosol amended with low- or high-P treatments. Shown are shoot P concentration as a % shoot DW, ratio of root DW to shoot DW, total root length, ratio of total fine-root (<0.36mm dia.) length to total thick root (≥0.36mm dia.) length, average root diameters, and P-uptake efficiency (PUE) expressed as shoot P per unit root length (µg P/ m root) and per unit root DW (mg P/g root DW). Values are means (*n*=4) and LSD (Fisher Method) (P<0.05) are provided. The asterisks indicate significant differences to NIL_Tall_ within each P treatment.

Genotype	Shoot P (% DW)	DW ratio of root:shoot	Total root length (m)	Root length ratio of fine:thick	Root Diameter (mm)	PUE_Length_ (µg P m^–1^)	PUE_Root DW_ (mg P g^–1^ DW)
**Low P**							
NIL_TALL_	0.313	0.454	44.32	3.07	0.35	33.2	7.0
NIL_SD_	0.340	0.492	33.87*	3.23	0.33	32.5	7.1
NIL_DWF_	0.315	0.515	33.86*	2.97	0.33	27.9	6.2
LSD	NS	NS	7.27	NS	NS	NS	NS
**High P**							
NIL_TALL_	0.439	0.267	36.72	2.26	0.37	110.6	17.6
NIL_SD_	0.470	0.274	36.38	2.47	0.36	102.4	17.8
NIL_DWF_	0.454	0.307	29.25	2.19	0.37	91.1*	15.3
LSD	NS	NS	NS	NS	NS	9.9	NS

## Discussion

### QTLs for shoot biomass on low-P soil

We identified QTLs for shoot biomass on a low-P soil in two biparental populations of wheat. Seven significant QTLs were identified from the Chuan-Mai 18/Vigour 18 RILs. The largest from Vigour 18, on chromosome 7A, accounted for 7.4% of phenotypic variation. Other QTLs accounting for >4% of variation were on chromosomes 3B and 4D (Chuan-Mai 18) and chromosome 4A (Vigour 18). Biomass was not correlated with tiller number in this study, and none of the QTLs mapped to chromosome 6A where a QTL for leaf width was identified previously in the same population (Spielmeyer *et al.*, 2007) or with the gibberellin-sensitive dwarf allele *Rht8* on chromosome 2D (Chuan-Mai 18). For the Kukri/Janz population, nine significant QTLs were identified, which accounted for 54.3% of the total phenotypic variance. Only chromosome 1B had a QTL from both populations, but it is unclear whether these co-located. Two QTLs on chromosomes 4B and 4D from the Kukri/Janz population collectively accounted for 24.8% of the variance, while others from Janz (chromosome 2DL) and Kukri (chromosome 6A) accounted for 7.8 and 7.1% of the phenotypic variance, respectively. Candidates underlying the QTLs on chromosomes 4B and 4D are the *Rht* alleles *Rht-D1b* (Kukri) and *Rht-B1b* (Janz). We confirmed the probable influence of *Rht* genes on the distribution in Fig. 1B by comparing NILs with different *Rht-1B* alleles at high and low P. Although these NILs had a different background (Maringa), they allowed us to directly compare the effect of the *Rht-B1* alleles on P uptake and efficiency in isolation from the other genetic factors. These experiments were successful and achieved two outcomes: (i) they confirmed the probable contribution of the *Rht* genes to the biomass distribution from the original DH screen; and (ii) they allowed us to quantify the early effects of the *Rht-B1* alleles on root growth and P efficiency in controlled conditions. These could not be done so easily in the population, which is segregating for many other traits.

Some QTLs for traits associated with P deficiency and P responsiveness have been reported previously in wheat. For instance, QTLs for greater biomass per unit P absorbed were reported on chromosomes 1B, 2B, 2D, 3B, 5A, and 7A from a set of RILs from W7984 and Opata85 screened in hydroponics with high- and low-P levels (Weidong *et al.*, 2001). Later studies by Su *et al.* (2006, 2009) screened two doubled-haploid populations in pot and field trials. These results implicated tiller number, shoot biomass per unit P uptake (inverse of P concentration), and vernalization genes as potentially important components of P efficiency. QTLs common to both doubled-haploid populations clustered on chromsomes 4B, 5A, and 5D (Su *et al.*, 2006, 2009). QTLs on these chromosomes were also identified in the present study, but further investigation is required to show whether the genomic regions are the same.

Interactions between dwarfing genes and P nutrition have received relatively little attention. Manske *et al.* (2002) measured P uptake and grain yield in high P for two sets of NILs differing in *Rht* alleles and found that the recurrent background was an important factor. In that study, the semi-dwarf alleles *Rht-B1b* and *Rht-D1b* had no effect on final shoot biomass but were associated with greater total P uptake at tillering and anthesis and with greater PUE at maturity (defined as grain biomass per unit P in the aboveground biomass). The present study found that the *Rht-B1b* and *Rht-B1c* alleles significantly reduced shoot biomass compared with the tall line (*Rht-B1a*) in high- and low-P treatments.

*Rht* genes can also affect root growth, although the reported responses vary. An early study found no differences in rooting depth between three semi-dwarf wheats and tall wheats (Cholick *et al.*, 1977). Miralles *et al.* (1997) later compared tall, semi-dwarf, and double-dwarf (*Rht-B1b*+*Rht-D1b*) genotypes and found that dwarf lines had more root length and root biomass in field-grown plants at anthesis. Wojciechowski *et al.* (2009) found that total root length was reduced in dwarf plants (*Rht-B1c* and *Rht-D1c*) but not in semi-dwarf plants grown in pots and in the field. By contrast, no significant correlations were detected between *Rht* genes and root biomass or root depth in an association mapping panel of 250 wheat cultivars (Narayanan and Prasad, 2014). Two studies that compared root traits in young seedlings grown in paper cigar rolls also came to different conclusions. The first study scored 159 RILs segregating for *Rht-B1b* and concluded that tall plants had 5–10% greater total root length depending on the growth temperature (Li *et al.*, 2011). The other study showed that *Rht-B1c* and *Rht-D1c* reduced total root length compared with controls, and that *Rht-D1b*, *Rht-B1c*, and *Rht-D1c* reduced root biomass but not root-to-shoot ratios (Bai *et al.*, 2013). Furthermore, total root length and total fine-root length were significantly reduced in the dwarf lines *Rht-B1c* and *Rht-D1c*. Our results are generally consistent with some findings from Wojciechowski *et al.* (2009) and Bai *et al.* (2013), since NIL_SD_ (*Rht-B1b*) and NIL_DWF_ (*Rht-B1c*) plants had less total root length than NIL_Tall_ plants at low P but not high P, and no genotypic differences in total root biomass, root diameter, or root-to-shoot ratios at high P were found. However the responses of root fractions to P treatment did differ between the NILs. Fine-root length in NIL_Tall_ and NIL_DWF_ increased in low P compared with high P, but the total length of thicker roots was unaffected. By contrast, fine-root length in NIL_SD_ plants was unaffected by P treatment but the length of thicker roots was smaller in low P. Experimental differences and environmental interactions will explain some of the variability between current and previous reports. Our results are consistent with the previous reports showing that the dwarf and semi-dwarf alleles reduced total root length in pot experiments but had smaller effects on total root biomass in fertilized pot trials (Miralles *et al.*, 1997; Wojciechowski *et al.*, 2009) and field-grown plants (Manske *et al.*, 2002). However, it is clear from mutant studies that DELLA proteins are important in roots and, for instance, generate signals that regulate symbiotic relationships. DELLA proteins are essential for nodule formation in pea (*Pisum sativum*) because the *la cry-s* mutant, which lacks DELLA proteins, formed significantly fewer nodules than wild-type plants (Ferguson *et al.*, 2011). Similarly DELLA proteins are also required for arbuscule formation by mycorrhizal fungi in *Medicago truncatula*. Arbuscule formation was severely impaired in the *Mtdella1/Mtdella2* double mutant, which is defective in DELLA synthesis (Floss *et al.*, 2013). These studies demonstrate that DELLA proteins not only function in roots but are essential for signalling pathways involved in establishing symbiotic relationships.

### Early vigour and P uptake

Our results indicated that early vigour contributed to biomass accumulation at low P in both populations examined. For the Chuan-Mai 18/Vigour 18 population, this conclusion was based on two results: (i) RILs from the upper tail of Fig. 1A had larger embryos and wider leaves than RILs from the bottom tail, and both these characteristics have previously been correlated with vigour (Lopez-Castaneda *et al.*, 1996; Rebetzke *et al.*, 2008); (ii) when RILs from the upper and lower tails were grown on high, non-limiting P, a significant difference in biomass was maintained. However, since the biomass of the tails was closer in high-P than in low-P conditions, we conclude that variation in the capacity to access P also influenced the distribution in Fig. 1A. This was confirmed in subsequent experiments with AVLs. The AVLs were chosen to test the role of vigour on P nutrition because an early vigour trait was the dominant feature of that material, which was derived, in part, from Vigour 18. It also allowed us to perform a more detailed characterization of the roots than was completed in the initial large screens. The use of this material could also be viewed as confirmation that vigour is important, since it included different genetic backgrounds.

These AVLs not only accumulated more biomass at low P but also had lower shoot P concentrations and greater PAE and PUE_Length_. These findings support previous suggestions that more biomass with a lower P concentration is a promising combination for improving the P-use efficiency of wheat (Su *et al.*, 2006). The ability to generate more biomass with less P is indicative of another definition of P-utilization efficiency (Rose *et al.*, 2013). The cause of the correlation between embryo size and vigour is unknown, but perhaps larger embryos have more resources readily available for germination and early growth and vigour. The larger embryos in this material are not necessarily a desirable trait if this reduces flour extraction from the total grain, but this effect was found to be relatively small in a mapping population varying for these traits (Moore, 2004).

Early vigour also contributed to the biomass distribution in the Kukri/Janz population because candidates underlying two large QTLs are *Rht-1* genes. These genes encode DELLA proteins, which function as growth suppressors throughout the life cycle of vascular plants The cellular content of DELLA proteins is regulated by degradation pathways involving binding of DELLA to the complex formed between gibberellin and its receptor (Hirano *et al.*, 2010). The semi-dwarf allele *Rht-B1b* results from a mutation that is proposed to result in an N-terminally truncated DELLA protein that is not subject to degradation. This affects cell length and coleoptile and plant height, and generally suppresses early vigour (Allan, 1989; Keyes *et al.*, 1989; Miralles *et al.*, 1998; Botwright *et al.*, 2001). The dwarf habit of *Rht-B1c* results from an in-frame insertion of 30 aa, which results in a DELLA protein that no longer binds to the receptor–gibberellin complex, and presumably accumulates to higher-than-normal levels (Pearce *et al.*, 2011). Two other QTLs from the Kukri/Janz population on chromosomes 2D and 6A collectively represent 16% of the phenotypic variation and warrant further investigation.

An early vigour-like phenotype has been described in transgenic plants expressing vacuolar pyrophosphatase enzymes (Gaxiola *et al.*, 2002, 2011). Transgenic rice and barley lines expressing the *AVP1* gene from *Arabidopsis* have significantly larger roots and shoots than null plants in control conditions (~60% more biomass) (Yang *et al.*, 2007; Schilling *et al.*, 2014). Interestingly, these plants typically grow larger under limited phosphate and nitrogen supply than controls and show greater tolerance to water stress and salinity (Yang *et al.*, 2007; Gaxiola *et al.*, 2011, 2012; Schilling *et al.*, 2014). AVP1 proteins hydrolyse inorganic pyrophosphate and pump protons across the tonoplast, but exactly how this transgene generates such a diverse range of phenotypes remains unclear. The generation of more acidic vacuoles could energize transport processes across the tonoplast or the increased hydrolysis of pyrophosphate could relieve inhibition of gluconeogenesis (glucose production using non-carbohydrate substrates). Alternatively changes to auxin or sugar transport could modify root structure and root function (Schilling *et al.*, 2014). Despite these uncertainties, these transgenic plants provide further examples linking early vigour with enhanced tolerance to low-nutrient conditions and other abiotic stresses.

Early vigour can be especially beneficial in Mediterranean-like climates since the rapid development of a large canopy shades the soil. This reduces evaporation, improves water-use efficiency (Leuning *et al.*, 1994; Lopez-Castaneda and Richards, 1994), and increases weed competitiveness (Coleman *et al.*, 2001). The early selections of Vigour 18 and other vigour material were made with these goals in mind (Rebetzke and Richards, 1999; Richards and Lukacs, 2002). Vigour 18 was subsequently shown to have superior nitrogen-use efficiency since it absorbed more nitrogen and accumulated more biomass than three other commercial cultivars in field trials and glasshouse experiments (Liao *et al.*, 2006).

The current results demonstrated that ‘early vigour’ is a blanket term to describe a general phenotype that can arise via different metabolic and physiological pathways. For example, in Vigour 18 and the AVLs, early vigour was positively correlated with embryo size and was independent of *Rht* genes. By contrast, vigour in the Kukri/Janz population was unrelated to embryo size but linked, in part, with *Rht* genes. Not surprisingly these diverse sources of vigour affect other aspects of plant biology in different ways, including P nutrition. This is clear from the present results. The AVLs showed altered root structure and greater PAE, PUE_Length_, and P-utilization efficiency than a less vigorous control. By contrast, the different *Rht* genes could affect biomass accumulation but not root structure, shoot P concentrations, PAE, or PUE. Therefore, while both sources of vigour increased overall plant size only the AVL material showed concomitant increases in PAE and PUE_Length_ and therefore provided extra benefits to growth at low P. These contrasting responses to P could be a direct consequence of the biochemistry generating the vigour phenotypes in each case, or they could be caused by additional traits linked to the vigour loci, which affect growth and nutrition in contrasting ways. We conclude that natural variation in early vigour can benefit P nutrition in cereals. This outcome highlights the value of alternative dwarfing genes that reduce plant height without affecting early vigour (Ellis *et al.*, 2004).

## Supplementary data

Supplementary data are available at *JXB* online.

Supplementary Table S1. Suggestive QTLs from the segregating populations.

Supplementary Fig. S1. Chromosomal locations of QTL for biomass for the Chuan-Mai 18/Vigour 18 wheat RIL mapping population.

Supplementary Fig. S2. Relationship between leaf width and shoot biomass of tail lines of the Chuan-Mai 18/Vigour 18 population grown at high P.

Supplementary Fig. S3. Embryo size of the AVLs and other genotypes.

Supplementary Fig. S4. Chromosomal locations of QTLs for biomass for the Kukri/Janz doubled-haploid mapping population.

Supplementary Fig. S5. Appearance of tillers on the NILs varying in *Rht* alleles.

Supplementary Fig. S6. Effect of *Rht* alleles on root growth on high- and low-P treatments.

Supplementary Data
