# Supplementary material for: Early vigour improves phosphate uptake in wheat
Source: J Exp Bot. 2015 Aug 28;66(22):7089–100. doi: 10.1093/jxb/erv403 (PMC4765783; doi:10.1093/jxb/erv403)
Supplement: Supplementary Data [file supp_66_22_7089__index.html]

Early vigour improves phosphate uptake in wheat — Early vigour improves phosphate uptake in wheat — Supplementary Data 

# Early vigour improves phosphate uptake in wheat

## Supplementary Data

Data files

- Supplementary Data - Supplementary Data
