# Supplementary material for: Early vigour improves phosphate uptake in wheat
Source: J Exp Bot. 2015 Aug 28;66(22):7089–100. doi: 10.1093/jxb/erv403 (PMC4765783; doi:10.1093/jxb/erv403)
Supplement: Supplementary Data [file supp_erv403_jexbot148304_file001.pdf]

SUPPLEMENTARY INFORMATION

Manuscript Title: Early vigour improves phosphate uptake in wheat.

Authors: Peter R. Ryan, Mingtan Liao, Emmanuel Delhaize, Gregory J Rebetzke, Chandrakumara Weligama, Wolfgang Spielmeyer, Richard A James

Table S1. Suggestive QTLs (genetic effect is significant at P = 0.10) from the segregating populations.
Estimated genetic (additive) effects, nearest linked molecular marker and chromosomal location for biomass QTL measured on 162 random doubled-haploid progeny from the Chuan-Mai 18/Vigour 18 (CM18/Vig18) and Kukri/Janz wheat populations. Positive additive effects indicate that the first parent allele (here Kukri and Chuan-Mai 18) is associated with increased biomass whereas a negative effect indicates Janz and Vigour 18 contributed the positive allele. The '*a*' the additive effect is estimated as one-half the difference in homozygotes carrying either parental allele (total phenotypic variance a/c was 71 and 70% for two experimental runs 1 and 2, respectively).

Chromosome	Nearest marker	QTL position (cM)[A]	*a* genetic effect (%)	Percentage phenotypic variance (σ^2_P)	LOD
CM18/Vig18					
2A	*nw1045*	106	0.06	2.6	2.9
2B	*nw2236*	121	0.05	2.7	2.7
3D	*nw2064*	127	-0.15	2.1	2.3
7A	*nw0245*	79	-0.09	2.9	2.7
Kukri/Janz					
2D	*gwm296*	24	0.05	2.6	2.7
4A	*nw1112*	165	0.07	3.0	2.6
5B	*gwm408*	117	0.05	1.9	2.6

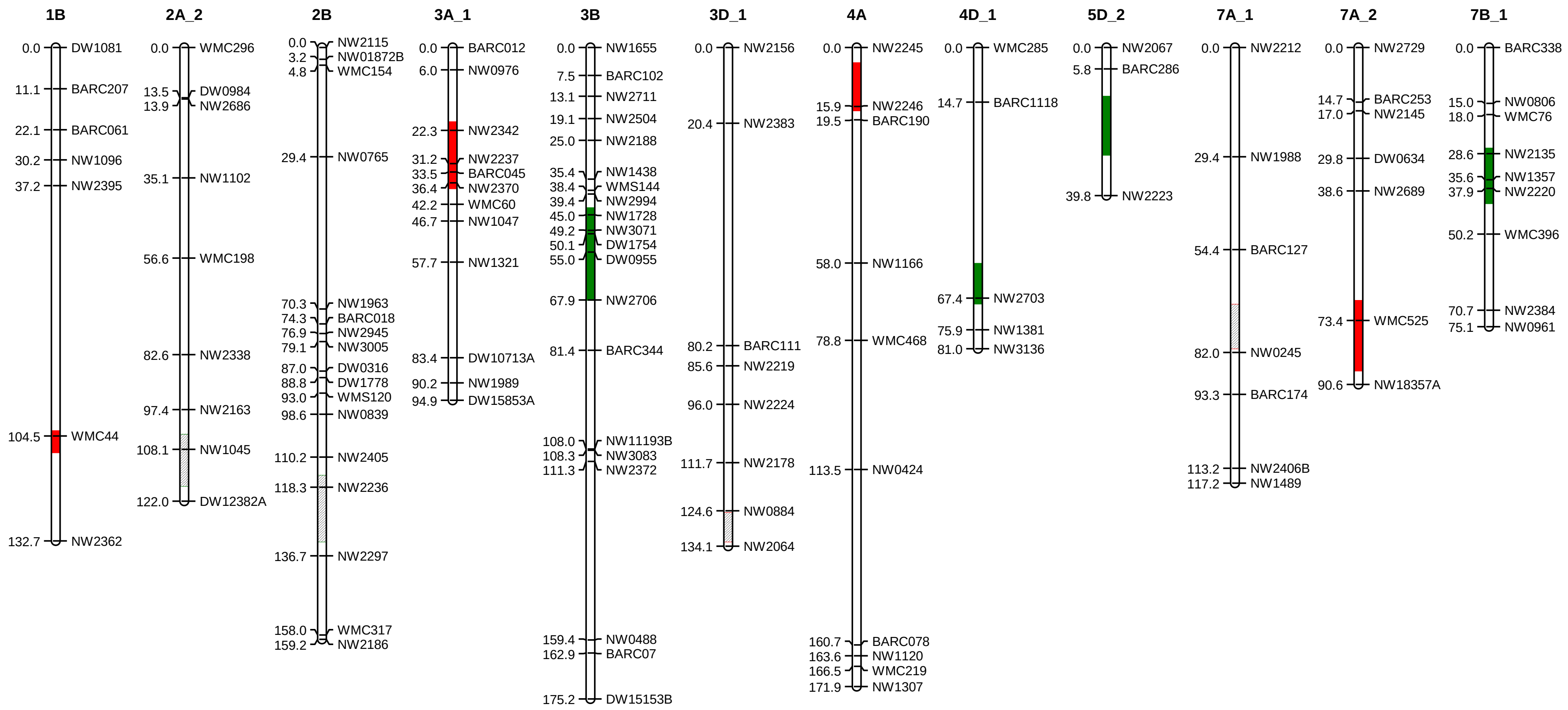

Supplementary Fig 1. Chromosomal locations of QTL for biomass in a soil low in plant-available P for the Chuan-Mai 18/Vigour 18 wheat RIL mapping population. Solid coloured bars indicate significant (P = 0.05 and 0.01) QTL with red and green colouring indicating negative and positive genetic effects respectively from the CM18 parent. Cross-hatched coloured regions are suggestive QTL (P=0.1).

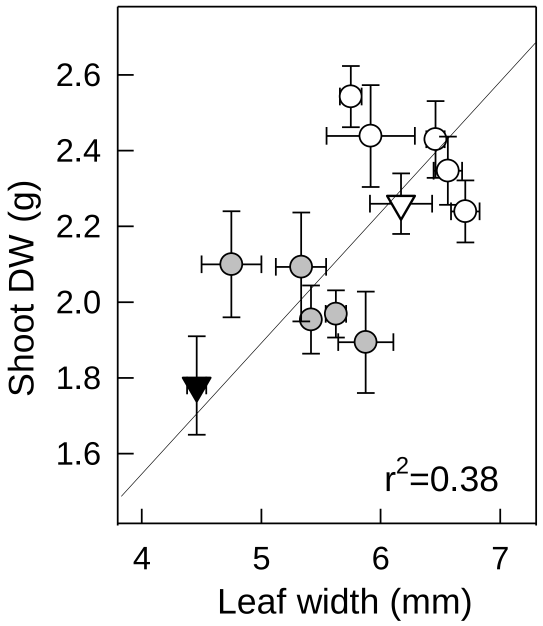

Supplementary Figure 2

Relationship between leaf width and shoot biomass of tails of the Chuan-Mai 18/ Vigour 18 population grown at high P. Leaf width was calculated as the average width of the first and second leaves measured at 27 DAS. Data show RILs from the lower tail (shaded circles) and upper tail (open circles). Mean and SE (where n=6).

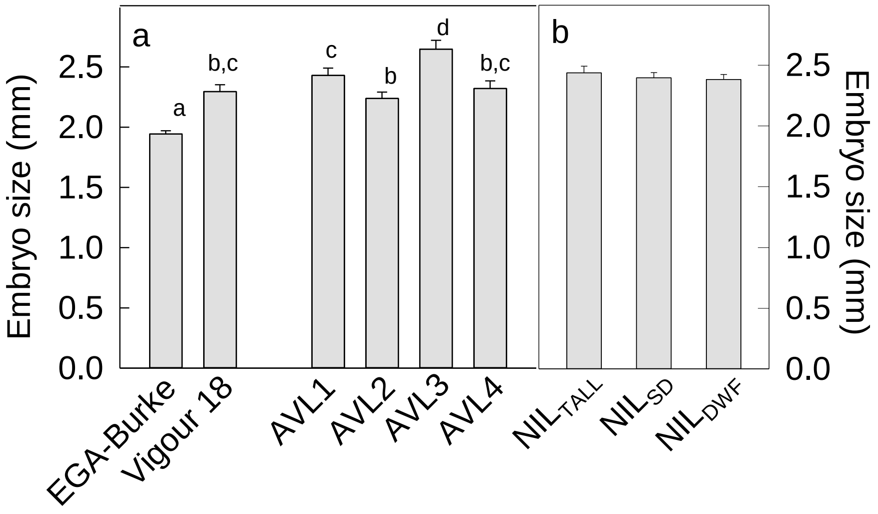

Supplementary Figure 3 Embryo size of the AVLs and other genotypes.

Relative embryo sizes were compared by measuring the width of the embryonic disc under a dissecting microscope. (a) Results from EGA-Burke, Vigour 18 and the AVLs. Data show means and SE (n=12 to 24). Different letters above each column indicate significant differences ($P<0.01$) based on a one way ANOVA where the LSD was 0.179. (b) Results from the Maringa NILs with different *Rht* alleles where NIL_{TALL}, NIL_{SD} and NIL_{DWF} represent *Rht-B1a*, *Rht-B1b* and *Rht-B1c* respectively. Data show means and SE (n= 20). There are no differences significant between the NILs.

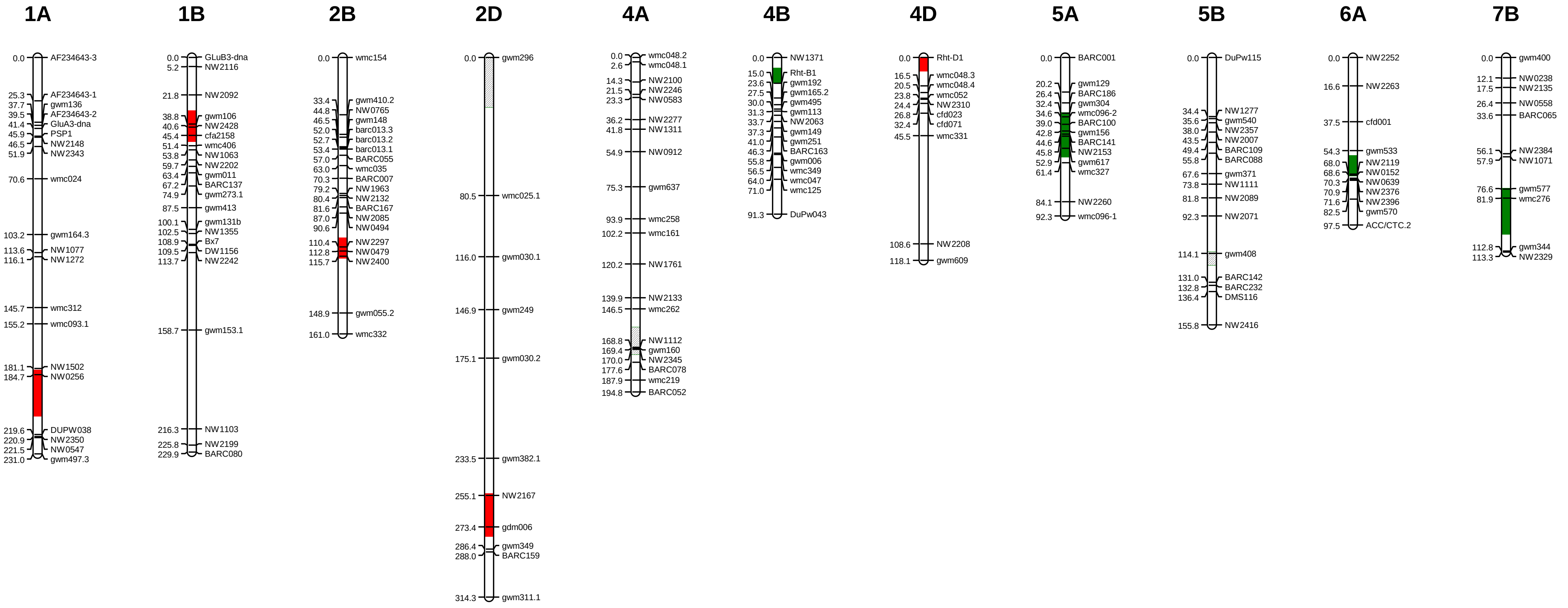

Supplementary Figure 4. Chromosomal locations of QTL for biomass in a soil low in plant-available P for the Kukri/Janz doubled-haploid mapping population. Solid coloured bars indicate significant (P = 0.05 and 0.01) QTL with red and green colouring indicating negative and positive genetic effects respectively from the Kukri parent. Cross-hatched coloured regions are suggestive QTL (P=0.1).

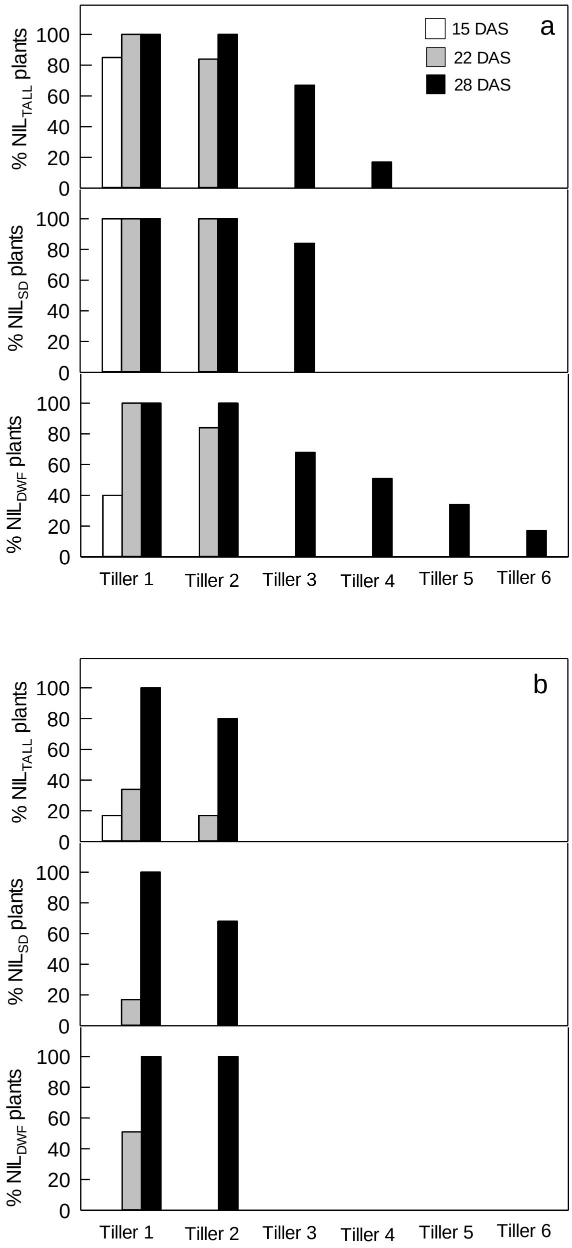

Supplementary Figure 5 Appearance of tillers on the NILs.

Results show the percentage of plants for each NIL with the indicated tiller number at 15, 22 and 28 DAS.

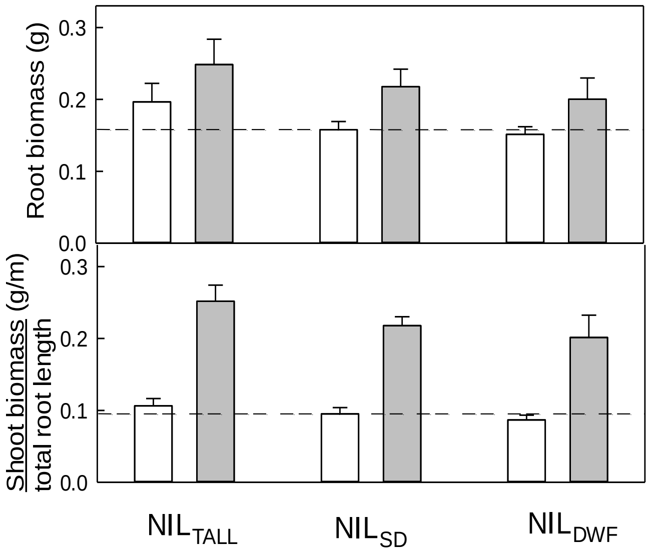

Supplementary Figure 6 Effect of dwarfing genes on root growth.

NILs were grown in soil amended with a low P (white bars) and a high, non-limiting P (shaded bars) treatments. Roots were harvested, washed and scanned with WinRHIZO^{TM}. Dashed lines indicate NIL$_{SD}$ at low P. Data show mean and SE (n=6). Two way analysis of variance were performed on these data found P treatment was significant ($P_{0.01}$, high P>low P) but genotype and the interactions were not significant.
